# Alcohol abuse as a potential risk factor of solitary death among people living alone: a cross-sectional study in Kyoto, Japan

**DOI:** 10.1186/s12889-022-12965-9

**Published:** 2022-03-19

**Authors:** Daisuke Miyamori, Tsukasa Kamitani, Yusuke Ogawa, Nozomi Idota, Hiroshi Ikegaya, Masanori Ito, Yosuke Yamamoto

**Affiliations:** 1 General Internal Medicine, Hiroshima University Hospital, Hiroshima, Japan; 2 Department of Forensic Medicine, Graduate School of Medical Science, Kyoto Prefectural University of Medicine, Kyoto, Japan; 3 Department of Healthcare Epidemiology, School of Public Health in the Graduate School of Medicine, Kyoto University, Kyoto, Japan

**Keywords:** Solitary death, Alcohol abuse, Socioeconomic status, Social isolation, Autopsy case

## Abstract

**Background:**

Solitary death is an emerging public health problem in developed countries. Alcohol abuse is associated with social isolation and excess mortality. However, data on the association between alcohol abuse and solitary death are limited. Our purposes were to assess whether alcohol abuse is associated with a long interval from death to discovery among people living alone.

**Methods:**

This is a cross-sectional study using the data on subjects from the largest forensic database in Kyoto, Japan, from February 2012 to December 2015. Solitary death was defined as a phenomenon of dying alone at home and remaining undiscovered for more than 1 week. All the subjects who lived alone and aged over 18 at the time of death were included in the study. The presence of alcohol abuse was identified via an investigation during home visits. Proportional ratios were calculated using a fitted logit model to evaluate the association of alcohol abuse on solitary death after adjusting for possible confounders.

**Results:**

A total of 235 subjects were included in the analysis. The mean age (standard deviation) of subjects at the time of death was 63.4 (15.1) years, and approximately 61.8% and 38.9% of subjects in the alcohol and non-alcohol abuse groups, respectively, experienced solitary death. Multivariable analyses revealed that alcohol abuse was associated with solitary death (adjusted proportion ratio: 1.50; 95% confidence interval: 1.12–2.00).

**Conclusions:**

The findings of this study could help identify individuals at higher risk of solitary death. Moreover, calling the attention of people with alcohol abuse may be beneficial to prevent solitary death.

**Supplementary Information:**

The online version contains supplementary material available at 10.1186/s12889-022-12965-9.

## Introduction

Japan is one of the countries with the highest population longevity. However, it also faces the challenge of an aging society. Recent studies have indicated that by 2060, two-fifths and one-fourth of the population will be aged over 65 and 75 years, respectively [1]. In addition, the proportion of individuals living alone in Japan has increased from 23.1% in 1990 to 32.6% in 2015 [2]. With these changes, in 1990 and 2015, there were approximately 14.7% and 21.1% of elderly individuals who lived alone, respectively [3].

According to the Japanese Ministry of Health, Labour and Welfare (MHLW), solitary death is considered as dying alone in the house, without the chance of going to a hospital, and remaining undiscovered after quite a while [4]. This phenomenon was first observed in the 1980s and was believed to have occurred only in Japan. However, it rapidly spread worldwide [5]. In the Tokyo metropolitan area, solitary death accounted for 6% of all deaths and 72% of unexpected deaths who lived alone, and the absolute number of solitary death between 2003 and 2016 increased from 1985 to 3190 for males and from 876 to 1414 for females [6]. Solitary death not only impairs the dignity of the deceased but also poses a substantial social and economic burden after death [7]. There are several intervention strategies for this phenomenon, such as letting people living alone to keep in touch with the community. However, optimal intervention strategies must still be established [8].

A previous study showed that living alone, unmarried status , male sex, loss of communication, old age, low physical activity, presence of non-communicable diseases (NCDs) and psychiatric disorders, low socioeconomic status (SES), and absence of relatives were considered the potential risk factors of solitary death among elderly individuals in Japan [9–13]. These factors were assessed independently rather than within the same analytical statistical framework. Thus, whether there is an association between solitary death and each risk factor after adjusting for potential confounding factors has not been confirmed.

Similarly, alcohol use disorder can cause social isolation and early mortality [14–16]. In Japan, the prevalence of alcohol use disorders was 1.9% for males and 0.2% for females in the general population, and the number of patients was approximately 1.07 million [17]. In terms of public health, MHLW declared to take measures in order to reduce harmful drinking in the national health promotion, “Healthy Japan 21” [18, 19]. Moreover, it is associated with psychiatric disorders and cardiovascular diseases, liver failure, osteoporosis, cognitive decline, and legal problems [20, 21]. This disorder could be a potential risk factor for solitary death. However, no data support this association.

This study aimed to assess the relationship between alcohol abuse and solitary death among individuals who experienced unexpected death. We hypothesized that prevalence of solitary death is higher in subjects with alcohol abuse compared to those without alcohol abuse.

## Materials and methods

### Study design

This is a cross-sectional study.

### Subjects

Subjects data were extracted from the largest forensic database in the Forensic Department of Kyoto Prefectural University of Medicine, Japan, from February 2012 to December 2015. All the individuals who died unexpectedly and were investigated in the department were recorded in the database. From the database, deceased individuals who lived alone were included in this study.

Unlike in western countries where the coroner system exists, the death investigation system in Japan is primarily governed by police [22]. Due to the lack of forensic physicians in Japan, not all unexpected death cases are investigated, and subjects with the unknown living situation were more likely to be under-investigated.

### Eligibility criteria

We set the time of death as the beginning of the observation period and the time interval from death to discovery as the primary outcome in the target population. Thus, subjects who had lived alone were the population at risk and those subjects were included in this study. We define individuals as living alone if they lived in their home without any partner, children, relatives, or acquaintances. Individuals below 18 years old and those living with their family were not included because they are under the responsibility of a guardian. Moreover, subjects with causes of death correlated with criminal cases, suicide, fire, automotive accidents, or drowning were excluded based on the definition of solitary death.

### Solitary death assessment

The outcome in this study was the prolonged time interval from death to discovery among subjects who lived alone. Solitary death was defined as a phenomenon of dying alone and remaining undiscovered for more than 1 week. The interval between death and discovery was confirmed based on the final judgment by forensic pathologists. Supporting information was investigated by the police officer.

### Presence of alcohol abuse

The presence of alcohol abuse was surveyed for all the deceased and confirmed based on the information obtained from the subject’s family physicians, neighbors, and relatives. Moreover, the police officer investigated the subject’s home regarding the possibility of crime and assessed their alcohol and food consumption, along with financial situations. Board-certified emergency physicians and forensic physicians were the responsible to interpret the presence of alcohol abuse.

### Potential confounding factors

Data on the subjects’ age, gender, a season of death (summer), smoking status, presence of NCDs and psychiatric disorders, existence of relatives, activities of daily living (ADLs), and SES status were collected. Since alcohol abuse is of interest in our study, alcohol-associated disorders were not included in the definition of psychiatric disorders.

Data on age, gender, existence of relatives, and welfare status were obtained from the census register. Smoking habits, presence of psychiatric disorders and NCDs, and status of performing ADLs were assessed using information obtained from the subject’s family physicians, neighbors, and relatives. If the deceased individual was on welfare, he/she was considered to have a low SES. Similarly, subjects who had any physical disability were considered to have low ADL. The time of death was confirmed in the death certificates.

### Statistical methods

Data were analyzed using Stata version 16.1 (StataCorp LP, College Station, TX). A *P-*value < 0.05 (two-tailed) was considered statistically significant. Only subjects without missing data were included in the statistical analysis. The subjects were classified into alcohol and non-alcohol abuse groups.

Data on the baseline characteristics between the alcohol and non-alcohol abuse groups were presented as mean and standard deviation for continuous variables and as number and proportion for categorical variables.

We used the method of Norton and colleagues to calculate proportional ratio (PR), a measure of the association between solitary death and alcohol abuse, because the frequency of the solitary deaths among the subjects was estimated to be higher than 10% [23]. First, we fitted three logit model: crude model, model adjusted with for age and sex, and the model adjusted for all potentiall confounders. Solitary death was considered a dependent variable. Second, mean predicted probability for alcohol abuse and non-alcohol abuse group on solitary death were converted from estimated coefficients in these logit models, through the formula of logistic cumulative distribution function. Finally, PR was calculated as ratio of the two mean predicted probabilities. We used the term PR rather than risk ratio because our study was based on prevalence. Instead of performing power analysis, the study period was determined to ensure that the number of events was greater than 10 per variable and the sample size was sufficient to conduct the study.

In the subgroup analysis, we evaluated effect modification in the association between alcohol abuse and solitary death. The subjects were classified into predefined groups based on factors such as SES, ADL, season of death, smoking habits, existence of relatives, and presence of psychiatric disorders and NCDs using the same model as in the primary analysis.

Two sensitivity analyses were performed to assess the association between the interval from death to discovery and alcohol abuse. First, we performed a multiple imputation procedure using the chained equation method on 180 cases with at least 1 missing confounding factor based on the missing-at-random assumption. The missing value was imputed using alcohol abuse and other confounding factors. We created 20 imputed datasets, which were analyzed using Poisson regression with a robust variance estimator. Then, the estimated results from each dataset were integrated [24]. Second, we used different postmortem intervals (1 day, 2 days, and 2 weeks) as outcome variables, and the adjusted PRs for each model were calculated after adjusting for the same confounding factors as those in the primary analysis.

### Ethical considerations

The study protocol was approved by the institutional review board of Kyoto Prefectural University of Medicine (ERB-C-615-1). The need to obtain informed consent was waived as this was a retrospective review of the subjects’ records according to the Japanese ethical guidelines for clinical research.

## Results

**Fig. 1:**
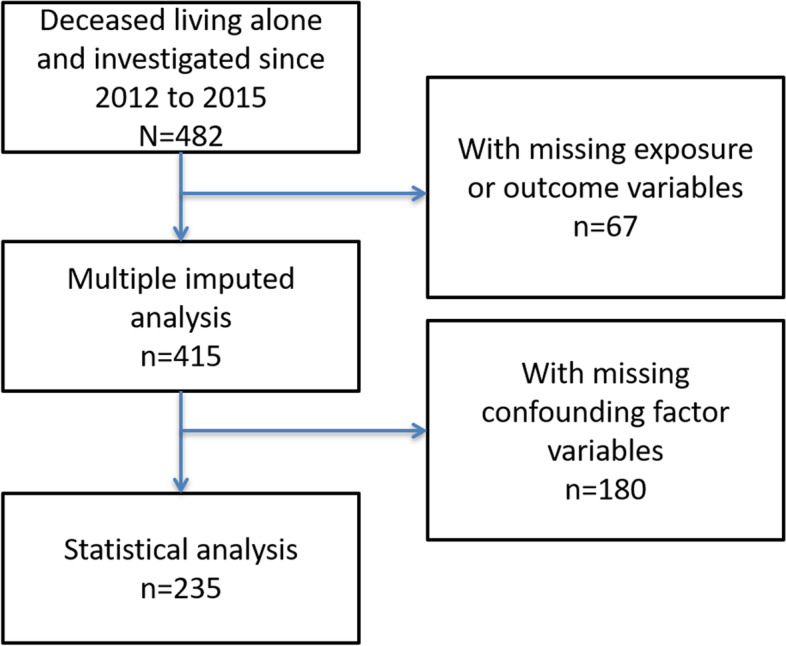
Flow chart for the selection of subjects

Figure 1 shows the number of qualified subjects. In this study, 482 cases were identified during the study period, and 415 were included in the analysis. Of these cases, 180 were excluded due to at least 1 missing value. Finally, 235 cases were included in the primary analysis.

Table 1 shows the baseline characteristics included in the statistical analysis. The mean age of the subjects in complete cases was 63.1 years, and men accounted for 72.8% of all participants. In complete cases, approximately 26.0% of participants presented with alcohol abuse. The mean age of the alcohol abuse group and the non-alcohol abuse group was 59.4 vs 65.0 years, respectively. The mean age of subjects with at least one missing value was 65.3 years, and men accounted for 67.8% of these subjects.

**Table 1:** Baseline characteristics of the subjects

	Total (*N* = 235)	Non-alcohol abuse (*n* = 167, 74.0%)	Alcohol abuse (*n* = 68, 26.0%)
Age, mean (SD)	63.4 (15.1)	65.0 (16.2)	59.4 (10.9)
Men, n (%)	172 (73.2)	111 (66.5)	61 (89.7)
Smoking habit, n (%)	101 (43.0)	54 (32.3)	47 (69.1)
Psychiatric diseases, n (%)	54 (23.0)	32 (19.2)	22 (32.3)
Any comorbidities, n (%)	159 (67.7)	114 (68.2)	45 (65.2)
Hypertension, n (%)	64 (27.2)	50 (29.9)	14 (20.6)
Diabetes, n (%)	33 (14.0)	24 (14.0)	9 (13.2)
Cardiovascular disease, n (%)	40 (17.0)	30 (18.0)	10 (14.7)
Cerebrovascular disease, n (%)	14 (5.9)	11 (6.6)	3 (4.4)
Kidney disease, n (%)	9 (3.8)	8 (4.8)	1 (1.5)
Cancer, n (%)	15 (6.4)	11 (6.6)	4 (5.9)
Cognitive decline, n (%)	11 (4.7)	10 (6.0)	1 (1.5)
Existence of relatives, n (%)	199 (84.7)	150 (85.6)	57 (82.3)
Summer season, n (%)	62 (26.4)	43 (24.6)	21 (30.9)
Low ADL, n (%)	31 (13.2)	23 (13.8)	8 (11.8)
Low SES, n (%)	67 (28.5)	35 (21.0)	32 (47.0)

*SD* standard deviation, *ADL* activities of daily living, *SES* socioeconomic status

### Association Between Alcohol Abuse and Solitary Death

Table 2 shows the PRs and 95% confidence intervals (CIs) for the association between alcohol abuse and solitary death. About 38.9% of participants in the non-alcohol abuse group and 61.8% in the alcohol abuse group experienced solitary death. Based on the results of the multivariate analysis, alcohol abuse was associated with a higher proportion of solitary death, compared with that of the reference group (crude PR: 1.59; 95% CI: 1.22–2.07, adjusted PR: 1.50; 95% CI: 1.12–2.00). The adjusted PRs for the other covariates are shown in Supplementary Table 1.

**Table 2:** Association between alcohol abuse and the potential risk factors of solitary death for complete cases

	Solitary death, n (%)	Proportion ratio (95% CI)		
		Crude	Age, Sex, Adjusted	Adjusted*
Alcohol abuse	42/68 (61.8)	1.59 (1.22–2.07)	1.57 (1.19–2.06)	1.50 (1.12–2.00)
Non-alcohol abuse (ref)	65/167 (38.9)	Ref	Ref	Ref

*CI* confidence interval, *ref* reference; *Adjusted for age, sex, socioeconomic status, smoking status, presence of psychiatric and noncommunicable diseases, existence of relatives, and activities of daily living

### Subgroup Analysis

The subgroup analysis results are shown in Table 3. There were missing values in terms of adjusted PR between the subgroups according to SES, ADL, season of death, smoking habit, presence of psychiatric disorders, and existence of relatives. The results were consistent with the main analysis in most subgroups except for the subgroup without relatives. The p-values for interaction were not significant between the alcohol abuse and each subgroup on solitary death. Supplementary Table 2 shows the analyses results for the same subgroups by changing the cutoff of the outcome into 2 weeks.

**Table 3:** Crude and adjusted Proportional Ratios of alcohol abuse on solitary death among subgroups of each potential risk factor

	Alcohol abuse		Non-alcohol abuse		Proportion ratio (95% CI)		*p*-value for interaction
	Solitary death	Total	Solitary death	Total	Crude	Age Sex Adjusted	
SES							0.33
Low	22	32	21	35	1.15 (0.80–1.63)	1.25 (0.86–1.83)	
High	20	36	44	132	1.67 (1.14–2.43)	1.60 (1.09–2.35)	
ADL							0.08
Low	6	8	5	23	3.45 (1.44–8.26)	4.44 (2.40–8.21)	
High	36	60	60	144	1.44 (1.09–1.91)	1.42 (1.07–1.89)	
Relatives							0.26
Present	35	56	52	143	1.72 (1.28–2.31)	1.67 (1.23–2.27)	
Absent	7	12	13	24	1.08 (0.59–1.97)	0.98 (0.48–2.00)	
Death in the summer season					0.84		
Yes	13	21	15	41	1.69 (1.00–2.86)	1.85 (1.11–3.08)	
No	29	47	50	126	1.55 (1.13–2.12)	1.46 (1.05–2.03)	
Smoking							0.34
Yes	27	47	21	54	1.48 (0.98–2.24)	1.48 (0.99–2.24)	
No	15	21	44	113	1.83 (1.29–2.62)	1.76 (1.21–2.56)	
Psychiatric diseases							0.30
Yes	11	22	12	32	1.33 (0.73–2.46)	1.48 (0.78–2.80)	
No	31	46	53	135	1.72 (1.28–2.29)	1.65 (1.22–2.23)	
NCDs							0.80
Yes	29	45	46	114	1.60 (1.17–2.18)	1.56 (1.12–2.16)	
No	13	23	19	53	1.58 (0.95–2.62)	1.56 (0.93–2.60)	

*CI* confidence interval, *ADL* activities of daily living, *SES* socioeconomic status, *NCDs* noncommunicable diseases

### Sensitivity Analysis

Meanwhile, the sensitivity analysis using multiple imputations showed that the crude PR, age- and sex-adjusted PR, and adjusted PRs for all confounding factors were 1.40 (1.14–1.71), 1.32 (1.07–1.62), and 1.25 (1.00–1.56), respectively. No marked differences from the main analysis were observed in the crude model and adjusted model using multiple imputations.

## Discussion

This cross-sectional study using the largest forensic database in Kyoto investigated the association between alcohol abuse and solitary death after adjusting for demographic, clinical, and social characteristics. In this study, alcohol abuse wasassociated with solitary death after controlling for potential confounding factors (adjusted PR 1.5, 95% CI; 1.12-2.00). Our results were robust both in the multiple imputation model and in the model adjusted for all potential confounding factors by changing the cutoff value of the outcome (Supplementary Table 3).

This study first reported the association between alcohol abuse and solitary death among unexpected death cases. A previous study showed that older age and male sex were the risk factors of solitary death. However, these potential risk factors were widely distributed and not changeable, thus it is difficult to focus on the population with these factors as a target of intervention [5, 9]. Therefore, this study was conducted to evaluate whether alcohol abuse is associated with solitary death. The result of our study was consistent with that of a previous study showing that death from hepatic diseases was associated with solitary death [9]. Although the etiology of hepatic diseases was not evaluated in the previous study, alcohol abuse might associate with the increase of solitary death because alcoholic fatty liver diseases had a higher prevalence and lower survival rate than other etiologies of hepatic diseases [25, 26]. In the multivariable adjusted model, other potential risk factors, such as low SES (adjusted PR 1.78, 95%CI 1.34-2.37) were also associated with the previous studies (Supplementary Table 1). These results suggested the importance of focusing on the presence of alcohol abuse in the risk assessment of solitary death.

Alcohol can be a significant risk factor for several medical conditions, from liver disease to cardiovascular diseases, infectious diseases, and malignancies [21]. In addition to the risk of excess mortality [15], alcohol abuse was found to be associated with a longer postmortem interval. One possible explanation is that individuals who abuse alcohol might lose the opportunity to keep in touch with society, communicate with others, including staff in medical institutions, and develop subsequent poor health behaviors and outcomes. This hypothesis is supported by a previous study, which showed that individuals with alcohol abuse experience family conflicts, withdraw from society, and present with psychological stress [27]. Notably, socially isolated individuals have a lower stress tolerance, which leads them to hazardous alcohol consumption [14–16]. Consequently, alcohol abuse and social isolation may reinforce each other. This study provided information about the possible mechanisms underlying the effect of alcohol abuse that have contributed to solitary death, including poor health behaviors and social isolation.

The subgroup analysis showed subjects with alcohol abuse was associated with solitary death in the most of subgroup, except for the subgroup without relatives, in which the age-sex adjusted proportional ratio was 0.98 (95% CI; 0.48-2.00). There was no significant interaction between alcohol abuse status and each subgroup category on the risk of solitary death. These results show the consistency and the robustness of the association between alcohol abuse and solitary death.

This study had several strengths. That is, we used a population-based database in a relatively large city where data of unexpected death cases were collected via the unified system. In this database, the subjects are not limited to patients from hospitals or registries for health checkups, which might minimize the potential source of selection bias. Since an isolated individual does not intend to respond to surveillance or interviews [28], nonresponse bias might have negatively influenced the questionnaire survey. Moreover, it is challenging to include participants with alcohol abuse in a prospective study and evaluate risk factors. A previous study showed that only 25% of individuals with alcohol use seek help or treatment [29]. Therefore, patients who did not receive standard care should be included in studies that assess the risk of solitary death.

This study also had several limitations that should be acknowledged. First, the diagnosis of alcohol abuse disorder might have caused misclassification bias. The diagnosis of this disorder is based on psychiatric consultations. Hence, alcohol abuse was considered an alternative indicator of an alcohol-associated behavior problem. However, alcohol consumption was challenging to estimate due to underreporting bias, particularly in the population with low SES [30–33]. In our study, alcohol abuse was confirmed by certified emergency physicians and forensic physicians using the information obtained by an expert police officer. This might be more precise than a self-reported diagnosis, particularly in terms of estimating alcoholic consumption.

Second, this study might have been affected by selection bias. Subjects in our study did not include all the population at risk who lived alone and died at their own home in Kyoto. This procedure was performed according to the unique investigation system of unexpected death in Japan, where subjects with the unknown living situation were more likely to be investigated in the forensic department. Thus, the target population of this study does not include all those who live alone, and the limitations of generalizability can be taken into account. However, in our study, we considered the population who lived alone and died at home as a risk population, and the characteristics of participants between this study and the previous ones are similar. Third, this is a cross-sectional analysis; therefore, we cannot address the causality or directionality of these associations. Finally, there are still possible mediating factors and confounding factors, such as frequencies of communicating with others, community involvement, perception of social loneliness, and availability of social support [34, 35]. The risk factors of solitary death may be modulated by several pathways including interpersonal, demographic, and medical factors. Therefore, further prospective research which includes the above potential risk factors are necessary to evaluate the causal inference between alcohol abuse and solitary death.

## Conclusions

In summary, we calculated proportional ratios from a fitted logit model using a large forensic database in Kyoto, Japan, to evaluate the association between alcohol abuse and solitary death. Alcohol abuse was significantly correlated with solitary death after adjusting for potential confounders. Results were consistent across the subgroup analyses and two sensitivity analyses of the model with different cutoffs of postmortem interval and the model with multiple imputations.

## Supplementary information

**Additional file 1.**

## Data Availability

The datasets generated and analyzed during the current study are not publicly available due to legal reasons but are available from the corresponding author, DM on reasonable request.

## Abbreviations

MHLW: Ministry of Health, Labour and Welfare
NCDs: Noncommunicable diseases
SES: Socioeconomic status
ADLs: Activities of daily living
PR: Proportional ratio
CIs: Confidence intervals
SD: Standard deviation
