# Supplementary material for: Alcohol abuse as a potential risk factor of solitary death among people living alone: a cross-sectional study in Kyoto, Japan
Source: BMC Public Health. 2022 Mar 19;22:545. doi: 10.1186/s12889-022-12965-9 (PMC8933924; doi:10.1186/s12889-022-12965-9)
Supplement: Supplementary file 1 — Additional file 1. [file 12889_2022_12965_MOESM1_ESM.docx]

| **Supplementary Table 1**  **Association between the potential risk factors and solitary death in a fully adjusted model** | | |
| --- | --- | --- |
|  | Adjusted proportion ratio* (95% CI) |  |
| Alcohol abuse | 1.50 (1.12–2.00) |  |
| Age (per 10 years) | 1.07 (0.92–1.24) |  |
| Male sex | 1.23 (0.86–1.76) |  |
| Low SES | 1.78 (1.34–2.37) |  |
| Low ADL | 0.70 (0.43–1.14) |  |
| Psychiatric diseases | 0.73 (0.49–1.09) |  |
| Existence of relatives | 0.98 (0.67–1.44) |  |
| Summer season | 1.02 (0.75–1.39) |  |
| Smoking | 0.80 (0.59–1.08) |  |
| NCDs | 0.99 (0.74–1.33) |  |
| ADL, activities of daily living; SES, socioeconomic status; NCDs, noncommunicable diseases; *Adjusted for age, sex, socioeconomic status, smoking status, presence of psychiatric and noncommunicable diseases, existence of relatives, and activities of daily living | | |

**Alcohol Abuse as a Potential Risk Factor of Solitary Death Among People Living Alone: A Cross-Sectional Study in Kyoto, Japan**

| **Supplementary Table 2**  **Proportional Ratios of alcohol abuse on postmortem interval greater than 2 weeks among subgroups of each potential risk factor** | | | | | | | | | | |
| --- | --- | --- | --- | --- | --- | --- | --- | --- | --- | --- |
|  | | Alcohol Abuse | |  | Non-Alcohol Abuse | | |  | Proportion ratio (95% CI) | |
|  | | Solitary death | Total |  | Solitary death | | Total |  | Crude | Age Sex Adjusted |
| SES |  | |  |  |  | |  |  |  |  |
| Low | 17 | | 32 |  | 16 | | 35 |  | 1.03 (0.61–1.72) | 1.02 (0.59–1.77) |
| High | 12 | | 36 |  | 27 | | 132 |  | 1.63 (0.92–2.89) | 1.60 (0.89–2.86) |
| ADL |  | |  |  |  | |  |  |  |  |
| Low | 4 | | 8 |  | 4 | | 23 |  | 2.87 (0.93–8.89) | 4.94 (2.18–11.2) |
| High | 23 | | 60 |  | 39 | | 144 |  | 1.42 (0.93–2.15) | 1.37 (0.90–2.09) |
| Relatives |  | |  |  |  | |  |  |  |  |
| Present | 24 | | 56 |  | 33 | | 143 |  | 1.86 (1.21–2.84) | 1.67 (1.23–2.27) |
| Absent | 3 | | 12 |  | 10 | | 24 |  | 0.60 (0.20–1.78) | 0.42 (0.13–1.41) |
| Death in the summer season | | |  | | |  | |  |  |  |
| Yes | 7 | | 21 |  | 6 | | 41 |  | 2.28 (0.88–5.92) | 2.68 (1.04–6.92) |
| No | 20 | | 47 |  | 37 | | 126 |  | 1.45 (0.94–2.22) | 1.33 (0.85–2.09) |
| Smoking |  | |  |  |  | |  |  |  |  |
| Yes | 20 | | 47 |  | 7 | | 54 |  | 1.77 (0.99–3.15) | 1.74 (0.98–3.07) |
| No | 7 | | 21 |  | 30 | | 113 |  | 1.26 (0.64–2.47) | 1.20 (0.59–2.41) |
| Psychiatric diseases | | |  | | |  | |  |  |  |
| Yes | 9 | | 22 |  | 5 | | 32 |  | 2.61 (1.01–6.76) | 2.90 (1.07–7.86) |
| No | 18 | | 46 |  | 38 | | 135 |  | 1.39 (0.89–2.18) | 1.32 (0.83–2.10) |
| NCDs |  | |  |  |  | |  |  |  |  |
| Yes | 21 | | 45 |  | 30 | | 114 |  | 1.77 (1.14–2.75) | 1.67 (1.06–2.65) |
| No | 6 | | 23 |  | 13 | | 53 |  | 1.06 (0.46–2.45) | 1.12 (0.48–2.62) |
| CI, confidence interval; ADL, activities of daily living; SES, socioeconomic status; NCDs, noncommunicable diseases | | | | | | | | | | |

| **Supplementary Table 3**  **Results of the sensitivity analysis for the association between alcohol abuse and different cutoff points for the outcome variables (n = 235)** | |
| --- | --- |
| Cutoff points for PMI | Adjusted proportion ratio* (95% CI) |
| >1 day | 1.03 (0.93–1.14) |
| >2 days | 1.19 (1.02–1.39) |
| >1 week | 1.47 (1.09–1.98) |
| >2 weeks | 1.35 (0.87–2.10) |
| CI, confidence interval; ORs, odds ratios; PR, proportion ratio; PMI, postmortem interval; *Adjusted for age, sex, socioeconomic status, smoking status, presence of psychiatric and noncommunicable diseases, existence of relatives, and activities of daily living | |

| **Supplementary Table 4**  **Proportional Ratios of solitary death on alcohol abuse among subgroups of each potential risk factor** | | | | | | | | | | | | |  |
| --- | --- | --- | --- | --- | --- | --- | --- | --- | --- | --- | --- | --- | --- |
|  | | Solitary death | | |  | Non-solitary death | | |  | | Proportion ratio (95% CI) | | p-value for interaction |
|  | | Alcohol-abuse | Total | |  | Alcohol-abuse | | Total |  |  | Crude | Age Sex Adjusted |  |
| SES | |  |  | |  |  | |  |  | |  |  | 0.65 |
| Low | | 22 | 43 | |  | 10 | | 24 |  | | 1.23 (0.70-2.14) | 1.35 (0.79-2.29) |  |
| High | | 20 | 64 | |  | 16 | | 104 |  | | 2.03 (1.14-3.62) | 1.96 (1.10-3.51) |  |
| ADL | |  |  | |  |  | |  |  | |  |  | NA |
| Low | | 6 | 11 | |  | 2 | | 20 |  | | 5.45 (1.32-22.6) | NA |  |
| High | | 36 | 96 | |  | 24 | | 108 |  | | 1.69 (1.09-2.61) | 1.67 (1.08-2.59) |  |
| Relatives | |  |  | |  |  | |  |  | |  |  | 0.21 |
| Present | | 35 | 87 | |  | 21 | | 112 |  | | 2.14 (1.35-3.41) | 2.03 (1.29-3.21) |  |
| Absent | | 7 | 20 | |  | 5 | | 16 |  | | 1.12 (0.44-2.87) | 0.98 (0.44–2.19) |  |
| Death in the summer season |  | | |  | | |  | | |  |  |  | 0.44 |
| Yes | | 13 | 28 | |  | 8 | | 34 |  | | 1.97 (0.96-4.07) | 2.21 (1.08-4.52) |  |
| No | | 29 | 79 | |  | 18 | | 94 |  | | 1.92 (1.16-3.18) | 1.73 (1.05-2.84) |  |
| Smoking | |  |  | |  |  | |  |  | |  |  | 0.51 |
| Yes | | 27 | 48 | |  | 20 | | 53 |  | | 1.49 (0.97-2.28) | 1.51 (0.98-2.33) |  |
| No | | 15 | 59 | |  | 6 | | 75 |  | | 3.17 (1.31-7.69) | 2.88 (1.20-6.91) |  |
| Psychiatric diseases |  | | |  | | |  | | |  |  |  | 0.50 |
| Yes | | 11 | 23 | |  | 11 | | 31 |  | | 1.35 (0.72–2.55) | 1.42 (0.78–2.57) |  |
| No | | 31 | 84 | |  | 15 | | 97 |  | | 2.38 (1.39-4.11) | 2.21 (1.29-3.80) |  |
| NCDs | |  |  | |  |  | |  |  | |  |  | 0.98 |
| Yes | | 29 | 75 | |  | 16 | | 84 |  | | 2.03 (1.20-3.43) | 1.89 (1.13-3.14) |  |
| No | | 32 | 19 | |  | 10 | | 44 |  | | 1.78 (0.90-3.55) | 1.77 0.89-3.52) |  |
| CI, confidence interval; ADL, activities of daily living; SES, socioeconomic status; NCDs, noncommunicable diseases; NA, not applicable | | | | | | | | | | | | |  |

**Supporting information**

Supplementary Table 1. Association between the potential risk factors and solitary death in a fully adjusted model

Supplementary Table 2. Proportional Ratios of alcohol abuse on postmortem interval greater than 2 weeks among subgroups of each potential risk factor

Supplementary Table 3. Results of the sensitivity analysis for the association between alcohol abuse and different cutoff points for the outcome variables (n = 235)

Supplementary Table 4. Proportional Ratios of solitary death on alcohol abuse among subgroups of each potential risk factor
